# HIV pre‐exposure prophylaxis initiation in community safe spaces increases PrEP access among key populations in Zambia

**DOI:** 10.1002/jia2.26487

**Published:** 2025-05-19

**Authors:** Linah K. Mwango, Caitlin Baumhart, Brianna Lindsay, Pawel Olowski, Henry Sakala, Chimika Phiri, Cassidy W. Claassen, Marie‐Claude C. Lavoie

**Affiliations:** ^1^ Ciheb Zambia Lusaka Zambia; ^2^ Center for International Health, Education, and Biosecurity University of Maryland School of Medicine Baltimore Maryland USA; ^3^ Institute of Human Virology University of Maryland School of Medicine Baltimore Maryland USA; ^4^ Maryland Global Initiatives Corporation Zambia Lusaka Zambia; ^5^ Zambia Key Population Consortium Lusaka Zambia; ^6^ Ministry of Health Lusaka Zambia; ^7^ Division of Global Health Sciences Department of Epidemiology and Public Health University of Maryland School of Medicine Baltimore Maryland USA

Comprehensive HIV prevention initiatives in Zambia are significantly blunting the epidemic spread. Between 2010 and 2021, new HIV acquisitions decreased by >50% [1], due to increased access to antiretroviral therapy (ART), comprehensive prevention and scale‐up of pre‐exposure prophylaxis (PrEP) since 2018. With these advancements, Zambia is nearing HIV epidemic control, with 88.7% of people living with HIV aware of their status; 98.0% of those are on ART, 96.3% of whom are virally suppressed [2].

Yet, 1.5 million Zambians remain at high risk of acquiring HIV, with about 810,000 ever‐initiating PrEP. Oral PrEP decreases the risk of HIV acquisition by more than 90% [3, 4, 5], poses few safety risks, and unlike barrier methods, can be taken discreetly and independently [6]. As of 2023, over four million people initiated PrEP globally [4, 7]. While sub‐Saharan Africa represents over 70% of the global HIV burden, the region only accounts for 44% of global PrEP initiations, with Zambia contributing just 9% [1, 7, 8].

Key populations (KPs)—men who have sex with men (MSM), female sex workers (FSW), transgender persons (TG) and people who inject drugs (PWID)—are at disproportionate risk for acquiring HIV [7] but are more likely to experience barriers to accessing PrEP due to stigma, criminalization and discrimination [8]. To address these gaps, the University of Maryland Baltimore's CDC‐funded Community Impact to Reach Key and Underserved Individuals for Treatment and Support (CIRKUITS) project developed a differentiated service delivery model for community‐based HIV prevention for KPs, which included:

1. **Integration of community prevention health posts with local health facilities**. In collaboration with local KP civil society organizations (CSOs), CIRKUITS mapped “hotspot” locations frequently accessed by KPs (guest houses, night clubs, bars). Based on hotspot data, we established community prevention health posts at key physical locations. Each health post is linked to the Ministry of Health (MOH) area health facility for supply chain management and data reporting via national health information systems. To ensure continuity of care, this linkage also enables seamless referrals for follow‐up services not available on‐site.
2. **Multidisciplinary teams**. Community prevention health posts are staffed by HIV nurse prescribers, community liaison officers, community health workers (CHWs), and monitoring and evaluation (M&E) officers. The nurse provides PrEP services and community initiations, manages refills, follow‐ups and referrals, and mentors other staff. Gatekeepers from local KP‐CSOs recruit peer KP‐CHWs and identify safe venues for KP services, while raising awareness about PrEP. Community liaison officers, trained in psychosocial counselling or social work, oversee the day‐to‐day activities of KP‐CHWs and provide individualized case management. CHWs conduct community outreach and organize peer support groups. CHWs are also MOH‐certified HIV testers and assist with home delivery of PrEP medication for selected hard‐to‐reach clients. M&E officers assist with clinical documentation and reporting requirements.
3. **Training all staff to deliver KP‐friendly, confidential and quality services**. All staff receive specific training to provide welcoming and non‐stigmatizing services to KPs. Nurses undergo training on KP‐sensitive care and PrEP clinical management, while community liaison officers are trained on KP sensitivity, safety and security, and community mobilization. CHWs complete a 6‐week training programme covering psychosocial counselling, health promotion, HIV risk assessment, HIV rapid testing and KP‐specific health services. CHWs must pass the national competencies exam to receive their HIV tester certification. All staff complete training on data security and confidentiality.
4. **Community prevention health post infrastructure and referral system**. Staff provide HIV testing, psychosocial counselling, PrEP, sexual reproductive health services, peer‐support groups and condom/lubricant distribution. The health posts include clinical consultation rooms, a lab providing point‐of‐care testing for sexually transmitted infections and urinalysis, and communal space for peer‐delivered group counselling and fostering communication between clients and staff.  Integration with the MOH facilitates referrals for HIV treatment and additional prevention services such as cervical cancer screening and voluntary medical male circumcision.

The CIRKUITS community PrEP programme expanded from four safe spaces in two districts to 13 safe spaces in 12 districts between October 2020 and September 2022. The programme now has 194 staff members, including 154 KP‐CHWs, 13 community liaison officers and 27 nurses, in addition to 140 gatekeepers. CHW retention increased from 57% in year 1 to 82% in year 2 after implementing measures like standardized stipends and transport reimbursement.

From 1st October 2021 to 1st March 2023, among 6,583 individuals eligible for and willing to start PrEP, 6,567 (99.8%) initiated PrEP at prevention posts. Among KPs, TG had the highest PrEP uptake, with all 241 (100%) initiating PrEP. PrEP uptake was also high among FSW (3,254/3,262; 99.8%); MSM (2,674/2,681; 99.7%); and PWID (398/399; 99.7%) (Figure 1). PrEP initiation rates were consistently high across all KPs, age groups and provinces, with near 100% uptake. By region, the highest PrEP uptake was in Western Province; by age, PrEP uptake was the highest among persons aged 45 years and above.

**Figure 1:**
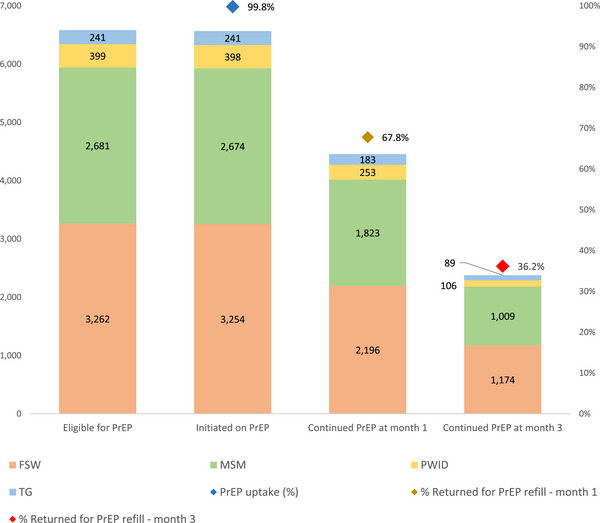
PrEP cascade by key population sub‐type.

For PrEP perseverance at month 1, 67.8% of initiated clients continued PrEP; this decreased to 36.2% at 3 months. Among KPs, TG had the highest PrEP continuation at 1 month (75.9%), while MSM had the highest continuation at month 3 (37.7%). PWID had the lowest PrEP continuation rates, at both 1 month (63.6%) and 3 months (26.6%).

The following lessons learned emerged:

1. **Integration of peer‐to‐peer model to reach KP**. Engaging trusted sources such as KP‐CSOs, KP gatekeepers and peer CHWs to reach KP is critical, especially given Zambia's restrictive laws related to homosexuality, transactional sex and drug use. Peer KP‐CHWs have been shown to increase HIV service uptake in Zambia [9, 10]. The peer‐delivered sessions and PrEP support groups in the CIRKUITS model increased PrEP awareness and knowledge while addressing common misconceptions. PrEP user support groups provided a forum to discuss key issues and support continued use, which encouraged clients to stay on PrEP.
2. **Engagement of community members from different KP groups**. Engagement with local KP‐CSOs is essential to reach marginalized groups in Zambia [9, 10], and they helped co‐design and effectively scale‐up the community prevention health post model. Their strategic guidance ensured services were tailored to reach marginalized KPs efficiently, and therefore, improved the availability and accessibility of services.
3. **Coordination across agencies working with KPs**. High CHW turnover initially disrupted services, leading to costs in new recruitment and training. The Zambia KP Consortium, a formal platform to discuss ongoing challenges and identify solutions, addressed this by establishing guidelines on CHW remuneration and working conditions to promote equity across organizations and reduce attrition.
4. **Effective MOH engagement**. Early engagement is critical and ensured alignment with national health priorities and sustained MOH support. Formal agreements and the involvement of frontline health workers fostered ownership and long‐term commitment for the programme providing free HIV biomedical prevention services.
5. **Data‐driven policy and sustainability**. Using data to highlight programme gaps and successes was key to advocating for expanded PrEP options, including injectable PrEP. MoH's involvement, particularly in placing healthcare workers at the posts, ensures accountability and long‐term sustainability.

We found community PrEP initiation through community prevention health posts to be an effective strategy for reaching underserved KPs in Zambia with biomedical HIV prevention interventions. However, PrEP persistence remained overall low, despite the implementation of multiple supportive strategies, including motivational interviewing regarding stigma and pill fatigue, peer support groups, flexible service delivery models like community‐based refills and phone consultations, and injectable PrEP at three sites. Further research is needed to understand factors influencing PrEP uptake and persistence, and to identify implementation strategies that support continued PrEP use.

## COMPETING INTERESTS

The authors declare that they have no conflicts of interest.

## AUTHORS’ CONTRIBUTIONS

LKM, CWC and HS conceived the project. LKM and CB conducted literature searches. BL and PO verified the source data and conducted data analysis. LKM, CB and M‐CCL wrote the initial draft. BL and CWC edited the manuscript and provided scientific and technical input. LKM, CB and CWC revised and finalized the manuscript. All authors have read and approved the final manuscript.

## FUNDING

The CIRKUITS project and this publication have been supported by the President's Emergency Plan for AIDS Relief (PEPFAR) through the U.S. Centers for Disease Control and Prevention (CDC) under the terms of NU2GGH002123.

## DISCLAIMER

The findings and conclusions in this report are those of the authors and do not necessarily represent the official position of the funding agencies.

## Data Availability

The data that support the findings of this study are available on request from the corresponding author. The data are not publicly available due to privacy or ethical restrictions.

## References

[1] UNAIDS . UNAIDS Country Data: Zambia. Accessed May 16, 2024. https://www.unaids.org/en/regionscountries/countries/zambia

[2] Centers for Disease Control and Prevention (CDC) . ZAMPHIA 2021 Summary Sheet. 2022. Accessed May 16, 2024. https://www.cdc.gov/globalhivtb/what‐we‐do/phia/ZAMPHIA‐2021‐Summary‐Sheet‐December‐2022.pdf

[3] Valente PK , Mantell JE , Masvawure TB , Tocco JU , Restar AJ , Gichangi P , et al. “I couldn't afford to resist”: condom negotiations between male sex workers and male clients in Mombasa, Kenya. AIDS Behav. 2020;24(3):925–937. 10.1007/s10461-019-02598-2 31321637 PMC6980499

[4] Van Damme L , Corneli A , Ahmed K , Agot K , Lombaard J , Kapiga S , et al. Preexposure prophylaxis for HIV infection among African women. N Engl J Med. 2012;367(5):411–422. 10.1056/NEJMoa1202614 22784040 PMC3687217

[5] Marrazzo JM , Ramjee G , Richardson BA , Gomez K , Mgodi N , Nair G , et al. Tenofovir‐based preexposure prophylaxis for HIV infection among African women. N Engl J Med. 2015;372(6):509–518. 10.1056/NEJMoa1402269 25651245 PMC4341965

[6] Bailey JL , Molino ST , Vega AD , Badowski M. A review of HIV pre‐exposure prophylaxis: the female perspective. Infect Dis Ther. 2017;6(3):363–382. 10.1007/s40121-017-0159-9 28600755 PMC5595773

[7] Periscopic. The Global PreP Tracker . A map‐based tool to explore trends in PrEP use globally as countries introduce and scale up PrEP programs. Accessed May 16, 2024. https://data.prepwatch.org/

[8] Kharsany ABM , Karim QA. HIV infection and AIDS in sub‐Saharan Africa: current status, challenges and opportunities. Open AIDS J. 2016;10:34–48. 10.2174/1874613601610010034 27347270 PMC4893541

[9] Mwango LK , Stafford KA , Blanco NC , Lavoie M‐C , Mujansi M , Nyirongo N , et al. Index and targeted community‐based testing to optimize HIV case finding and ART linkage among men in Zambia. J Int AIDS Soc. 2020;23(Suppl 2):e25520. 10.1002/jia2.25520 32589360 PMC7319128

[10] Mwango L , Toeque M , Lindsay B , Tembo K , Sakala H , Reggee S , et al. Reaching transgender populations in Zambia for HIV prevention and linkage to treatment using community‐based service delivery. J Int AIDS Soc. 2022;25(Suppl 5):e25995. 10.1002/jia2.25995 36225155 PMC9557009

